# The Emerging Roles of Ferroptosis in Vascular Cognitive Impairment

**DOI:** 10.3389/fnins.2019.00811

**Published:** 2019-08-06

**Authors:** Nao Yan, Jun-Jian Zhang

**Affiliations:** Department of Neurology, Zhongnan Hospital of Wuhan University, Wuhan, China

**Keywords:** vascular cognitive impairment, oxidative stress, lipid peroxidation, iron dyshomeostasis, ferroptosis

## Abstract

Vascular cognitive impairment (VCI) is a clinical syndrome that encompasses all forms of cognitive deficits caused by cerebrovascular disease, from mild cognitive impairment to dementia. Vascular dementia, the second most common type of dementia after Alzheimer’s disease (AD), accounts for approximately 20% of dementia patients. Ferroptosis is a recently defined iron-dependent form of cell death, which is distinct from apoptosis, necrosis, autophagy, and other forms of cell death. Emerging evidence suggests that ferroptosis has significant implications in neurological diseases such as stroke, traumatic brain injury, and AD. Additionally, ferroptosis inhibition has an obvious neuroprotective effect and ameliorates cognitive impairment in various animal models. Here, we summarize the underlying mechanisms of ferroptosis and review the close relationship between ferroptosis and VCI.

## Introduction

Vascular cognitive impairment (VCI) was first presented by Bowler in 1995, and this term seeks to identify cognitive impairment due to cerebrovascular disease at the very earliest stage and, by identifying the etiology, enables the institution of appropriate preventive therapy (Bowler and Hachinski, 1995). In recent years, VCI has been regarded as a more appropriate notion in describing a broad spectrum of cognitive and behavioral changes ranging from mild cognitive impairment (MCI) to dementia (Bowler, 2007; Frances et al., 2016). Indeed, VCI includes all the cognitive disorders associated with cerebrovascular disease and risk factors (Moorhouse and Rockwood, 2008). Vascular dementia (VD) accounts for about one-fifth of all dementia types, it is the second leading form of dementia next to AD and commonly coexists with AD (Iadecola, 2013; Price et al., 2018; Venkat et al., 2018). As a result of cognitive function decline, especially attention, information processing, and executive, this condition has become a heavy burden on individuals, families, and healthcare systems (O’Brien and Thomas, 2015; Dichgans and Leys, 2017). However, although great efforts have been made for many years, the pathologic mechanisms of VD are still poorly understood (Girouard and Munter, 2018).

The brain is a metabolic organ of high energy demands but does not have much energy reserve; it constitutes only 2% of total body mass but needs 15% of cardiac output and consumes 20% of the body’s oxygen and 25% of total body glucose. In addition, the brain is rich in lipids with unsaturated fatty acids, which are the key substrates for the production of lipid reactive oxygen species (ROS), thus the brain is more susceptible to oxidative stress than other organs via the imbalance of redox reaction (Dringen, 2000; Nagata et al., 2016; Cobley et al., 2018). The reduction of cerebral blood flow (CBF) resulting from vascular pathologies is the key contributor to cerebral redox imbalance. Meanwhile, chronic cerebral hypoperfusion (CCH) due to persistent decrease of CBF could result in cognitive impairments (Iadecola, 2013; Choi et al., 2016; Back et al., 2017). Oxidative stress is one of the main theories to explain the pathological mechanism of VCI (Du et al., 2017). However, how oxidative stress causes neuronal loss and subsequent neurodegeneration has not been fully elucidated. Recently, ferroptosis, a novel form of cell death, has been known to play an essential role in oxidative stress and neurological diseases (Dixon et al., 2012). It has begun to attract increased attention owing to its implication in several pathophysiological contexts. Here, we review recent studies on ferroptosis and discuss the close relationship between ferroptosis and VCI.

## Ferroptosis

**FIGURE 1:**
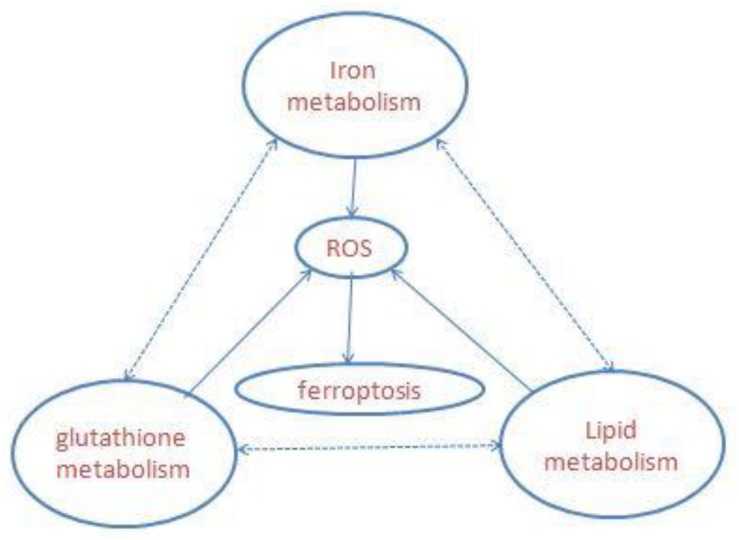
Overview of ferroptosis; the three central pathways regulate ferroptosis: iron, lipid, and glutathione metabolism, the synergistic effect of the three is to maintain the redox equilibrium under normal conditions. One of these metabolic disorders can lead to the accumulation of lipid ROS, then trigger ferroptosis.

Ferroptosis is a newly defined iron-dependent form of cell death which is morphologically, biochemically, and genetically distinct from apoptosis, necrosis, autophagy, and other forms of cell death. The symbol of ferroptosis is the accumulation of iron-induced lipid peroxidation, the depletion of glutathione (GSH), and inactivation of the phospholipid peroxidase glutathione peroxidase 4 (GPX4); this unbalanced redox triggers cell death (Yang W. S. et al., 2014; Stockwell et al., 2017; Figure 1). Interestingly, a novel compound, erastin, was first identified when the researchers screened antitumor agents. They found that erastin induced non-apoptotic cell death in order to kill engineered tumorigenic cells in 2003 (Dolma et al., 2003). They subsequently further screened out two Ras-selective lethal small molecular compounds (RSL3 and RSL5) that induced iron-dependent oxidative cell death in 2008. In addition, the iron chelator desferrioxamine (DFO) and the antioxidant (vitamin E) could prevent this form of cell death, which did not display apoptotic hallmarks (Yang and Stockwell, 2008). Consequently, the novel non-apoptotic cell death was termed “ferroptosis” in 2012 by Dr. Brent R. Stockwell and his team (Dixon et al., 2012; Alvarez et al., 2017). The morphological characteristic of ferroptosis is cell swelling, which is distinct from cell shrinking and blebbing during apoptosis. Ultrastructurally, mitochondria become smaller, with increased mitochondrial membrane density, reduced mitochondrial crista, and mitochondrial outer-membrane rupture (Xie et al., 2016; Angeli et al., 2017; Yu et al., 2017). Although the exact mechanism of ferroptosis is still poorly understood, ferroptosis regulation is closely associated with a variety of biological processes, mainly including iron, amino acid, and lipid metabolism (Yang and Stockwell, 2016).

## Iron Metabolism in Ferroptosis

**FIGURE 2:**
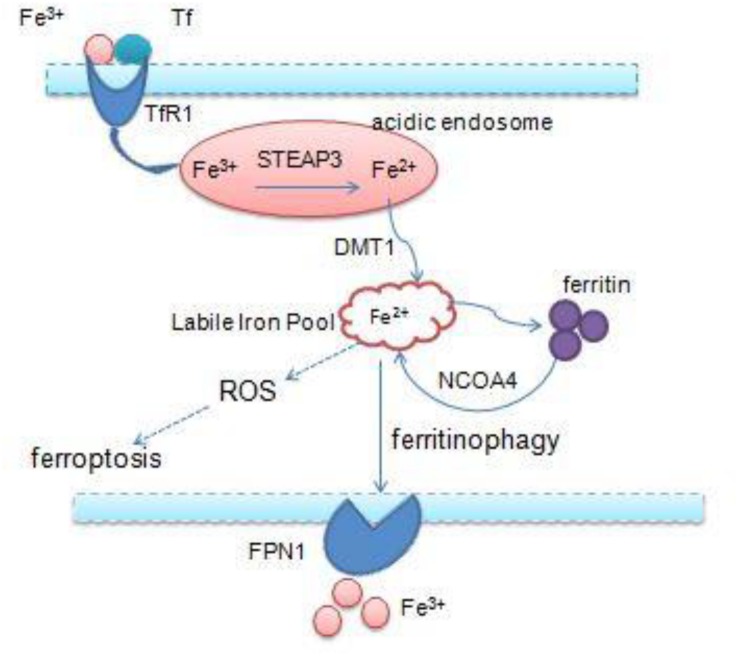
Overview of iron homeostasis: iron import, storage, export, utilization, and intracellular excess iron induce ferroptosis.

Iron is an essential transition metal for normal cellular function in mammals. It participates in several vital biological processes, including ATP generation, oxygen transport, and DNA synthesis. But excess intracellular iron can generate ROS via the Fenton reaction, which causes lipid peroxidation and cell death (Bogdan et al., 2016; Fanzani and Poli, 2017). Accumulation of intracellular free iron is the key to the execution of ferroptosis, and erastin/RSL3-induced ferroptosis can be inhibited by DFO, the iron chelator and its analogs (Fearnhead et al., 2017). Iron homeostasis is a complex process and relies on coordination of multiple mechanisms including iron import, storage, export, and utilization – therefore, a number of specialized transport systems and membrane carriers are essential (Singh et al., 2014; Figure 2). First of all, transferrin (Tf) and transferrin receptor1 (TfR1) are the main iron uptaking proteins, and cells take up iron mainly through the Tf–TfR1 pathway. Most of the plasma iron is bound tightly to Tf, which shields ferric iron (Fe3+) from redox activity; subsequently, they integrate with TfR1 on the cell surface, and the complex is taken up via endocytosis. Once inside the cell, ferric iron is released from Tf and free ferric iron is reduced to ferrous iron (Fe2+) by ferric reductase six-transmembrane epithelial antigen of prostate 3 (STEAP3) in the acidic endosome. Ferrous iron is thereby transported to the cytoplasm by divalent metal transporter 1 (DMT1) for their metabolic needs. DMT1, a metal transporter, is principally responsible for iron transport from the endosome (Ohgami et al., 2005; Ji and Kosman, 2015; Bogdan et al., 2016). Eventually, excess iron must be stored or exported across the plasma membrane ferritin (FTH1 and FTL), the iron–storage protein complex is mainly responsible for the sequestration of reactive iron in order to maintain the equilibrium of labile iron pool (LIP), thereby preventing the formation of ROS (MacKenzie et al., 2008). And iron export is mediated by the membrane protein ferroportin 1 (FPN1), which is the sole mammalian exporter transporting iron out of the cytosol (Troadec et al., 2010; Masaldan et al., 2019). In addition, recent studies have shown that autophagy also contributes to ferroptosis. Nuclear receptor coactivator 4 (NCOA4) is a selective cargo receptor, which mediates the autophagic degradation of ferritin. It indicates that NCOA4 binds to ferritin and delivers it to lysosomes for degradation, the process is termed “ferritinophagy.” NCOA4-mediated ferritinophagy increases intracellular iron level by releasing ferritin iron (Mancias et al., 2014; Gao et al., 2016; Santana-Codina and Mancias, 2018). NCOA4 deletion inhibited ferroptosis by blocking ferritinophagy and ferritin degradation, and NCOA4 over-expression increased sensitivity to ferroptosis; hence, autophagy contributes to ferroptosis by degradation of ferritin (Hou et al., 2016).

On the one hand, iron can catalyze the formation of free radicals from ROS via the Fenton reaction, then they can damage proteins, lipids, nucleic acids, and other cellular components, leading to cellular injury or death (Imam et al., 2017; Yu et al., 2017). On the other hand, iron is also an important component of the catalytic subunit of lipoxygenase (LOX), which is necessary for lipid peroxidation (Shintoku et al., 2017). Iron not only produces ROS directly, but also participates in the synthesis of iron-containing LOXs that oxidize polyunsaturated fatty acids (PUFAs) to result in lipid peroxides (Lei et al., 2019). Intracellular iron overload is the key to initiating ferroptosis. Although we know the close relationship between iron and ferroptosis, the specific molecular mechanism still needs further research.

## Lipid Metabolism in Ferroptosis

**FIGURE 3:**
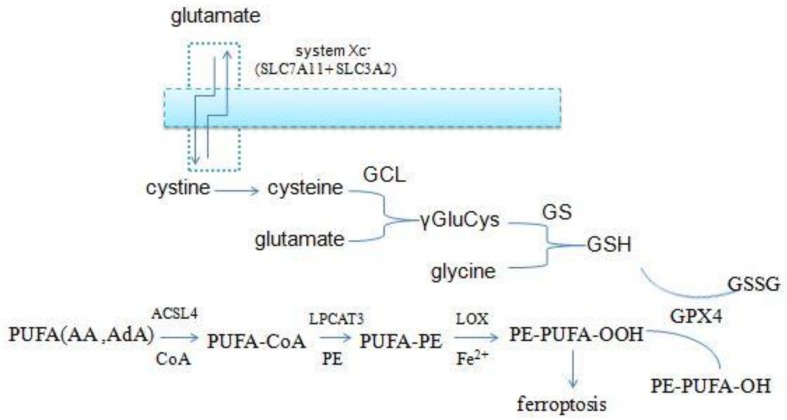
Overview of glutathione and lipid metabolism.

The accumulation of lipid ROS is the key process in initiating and executing ferroptosis, which is a complex process involving a lot of lipid metabolism productions (Magtanong et al., 2016; Figure 3). PUFAs, particularly arachidonic acid (AA) and adrenic acid (AdA), are the main substrates of lipid peroxidation for ferroptosis. They must be esterified with membrane phospholipids [mainly phosphatidylethanolamine (PE)] through two steps under the action of special enzymes (Hao et al., 2018). Acyl-CoA synthetase long-chain family member 4 (ACSL4), which is the catalytic enzyme for the first step, firstly catalyzes free AA/AdA to bind CoA to form AA/AdA–CoA derivatives, facilitating their esterification into phospholipids (Golej et al., 2011; Kuch et al., 2014). Next, lysophosphatidylcholine acyltransferase 3 (LPCAT3) catalyzes the biosynthesis of AA/AdA–CoA and membrane PEs to form AA/AdA-PE, which is an intermediate process to activate the ferroptotic signals (Shindou and Shimizu, 2009). ACSL4 is a member of a family of enzymes consisting of five isoforms comprising ACSL1, ACSL3, ACSL4, ACSL5, and ACSL6, but only ACSL4 specifically contributes to ferroptotic cell death and determines ferroptosis sensitivity (Yuan et al., 2016). Recent studies have revealed that inhibition of ACSL4 was effective in protecting against RSL3-induced cell death, suggesting that ACSL4 inhibition means a specific antiferroptotic pathway (Doll et al., 2016). Indeed, disruption of ACSL4 and LPCAT3 function has been shown to prevent ferroptosis (Dixon et al., 2015). Eventually, LOXs oxidate PE-AA/AdA to be PE-AA/AdA-OOH, identified as the cell death signal of ferroptosis. Lipid hydroperoxides were shown to be the proximate executors of ferroptosis. The research indicated that it was PE-AA/AdA-OOH rather than other types of phospholipids – OH (PL-OOH) – that induced ferroptosis (Lei et al., 2019). Recent research has revealed that LOXs, especially 15-LOX, have significant impacts on ferroptosis sensitivity. LOX-catalyzed lipid hydroperoxide generation in cellular membranes promoted ferroptosis, and several LOX inhibitors are cytoprotective in cell and animal models (Shintoku et al., 2017; Zilka et al., 2017; Shah et al., 2018), and 12-LOX is indispensable for p53-mediated ferroptosis (Chu et al., 2019). Accordingly, the lipid metabolism is tightly associated with ferroptosis and provides a promising theoretical pathway to prevent ferroptosis.

## Glutathione Metabolism in Ferroptosis

Glutathione peroxidase 4 is the unique antioxidant defense enzyme which reduces the membrane lipid hydroperoxides to lipid alcohols. Lipid hydroperoxides are detrimental products for ROS, thereby GPX4 can inhibit toxic lipid peroxidation (Cardoso et al., 2016; Cozza et al., 2017; Imai et al., 2017). Iron-dependent lipid peroxidation is the key step to triggering ferroptosis, and pharmaceutical inhibition and gene ablation of GPX4 function both can result in the accumulation of lipid ROS. Accordingly, GPX4 is the master regulator of ferroptosis (Friedmann Angeli et al., 2014; Yang W. S. et al., 2014; Conrad and Friedmann Angeli, 2015). However, GPX4 must use GSH as a cofactor to reduce peroxides to their corresponding alcohols. GSH is a small tripeptidyl molecule consisting of three amino acids, which alternates reduced (GSH) with oxidized (GSSG) states, thereby participating in redox biochemical reactions. GPX4 uses two molecules of GSH as substrates and produces one molecule of GSSG per cycle of catalysis, thus intracellular GSH levels are crucial to the activity of GPX4. Direct inhibition of GSH biosynthesis, or genetic manipulations, can trigger or sensitize to ferroptosis (Cao et al., 2019; Forcina and Dixon, 2019).

Intracellular cysteine is one of the raw materials for GSH synthesis. Most of the cysteine come from extracellular cystine via the cystine/glutamate antiporters (system xc-) which transport extracellular cystine and intracellular glutamate in a 1:1 ratio, then cystine is reduced to cysteine in cells. System xc- consists of two subunits (the light chain xCT and the glycosylated heavy chain 4F2hc) which are encoded by the SLC7A11 and SLC3A2 gene, respectively (Conrad and Sato, 2012; Dixon et al., 2014; Hayano et al., 2016). And the small molecule erastin, a potent inhibitor of the system xc-, can trigger endoplasmic reticulum (ER) stress and ferroptosis through depletion of GSH and loss of GPX4 activity (Dixon et al., 2014).

In addition to cysteine, glutamate and glycine are also intermediates for the synthesis of GSH. Firstly, cysteine and glutamate form the γ-glutamylcysteine (γGluCys) under the catalysis of the γ-glutamylcysteine ligase (GCL), then γGluCys continues to bind glycine to form GSH under the catalysis of glutathione synthetase (GS). The complete process consumes two molecules of ATP, with each step requiring one (Aoyama and Nakaki, 2013, 2015). Excitotoxicity of glutamate is also closely related to ferroptosis. Excessive extracellular glutamate can block system xc–mediated cystine import and results in GSH depletion and inactivation of GPX4 (Dixon, 2017). Iron chelation ciclopirox (CPX) and free radical scavengers ferrostatin-1 (Fer-1), two ferroptosis inhibitors, both can prevent cell death caused by excitotoxicity due to high glutamate, suggesting that the two cell death modes have a common mechanism. From the discussion above, it is suggested that the GSH metabolic network has a significant impact on ferroptosis (Figure 3).

## Links Between Ferroptosis and VCI

At present, VCI is considered a progressive disease caused by vascular diseases [e.g., cerebral small vessel disease (SVD)] and vascular risk factors (e.g., hypertension, hyperlipidemia, and diabetes), and SVD mainly caused cortical, subcortical, and lacunar microinfarcts due to pathological changes of perforating arteries and arterioles, capillaries, and venules. SVD is widely referred to as the most common vascular cause of VCI (Wardlaw et al., 2013; Kalaria, 2016, 2018). Indeed, VCI is a clinical syndrome that encompasses all forms of cognitive deficits, from MCI to dementia. VD, the second most common type of dementia after Alzheimer’s disease (AD), accounts for approximately 20% of dementia patients (Sun, 2018). Vascular abnormality is the leading pathology of VCI, which eventually causes cerebral hemodynamic alteration. Diverse vascular pathologies lead to chronic and significant decrease of CBF, including atherosclerosis, arteriolosclerosis, infarcts, white matter (WM) changes, and microhemorrhages (Calabrese et al., 2016; Yang T. et al., 2017). Thus, CCH caused by continuously reducing CBF is the common pathomechanism of VCI, which not only reduces the supply of oxygen and nutrients, but also leads to the accumulation and deposition of harmful molecules in the brain. The sustained low cerebral blood supply caused impairment of structure and function of the blood–brain barrier (BBB) and neuronal tissue loss and finally manifests as a cognitive deficit (Dichgans and Leys, 2017; Hort et al., 2019). So far, however, no effective treatments have been applied to prevent the progression of the disease, which has led to serious social burden (Kalaria, 2016; Venkat et al., 2018). Iron accumulation and excess oxidative stress cause cognitive impairment in aging and neurodegenerative diseases such as AD, Parkinson disease (PD), and Huntington’s disease (HD) (Ward et al., 2014). Iron-induced lipid peroxidation is the main characteristic of ferroptosis, and the research shows that ferroptosis plays a crucial role in neuronal loss of neurological diseases (Weiland et al., 2018). Researchers have recently clarified that neuronal ferroptosis is involved in ischemic stroke and intracerebral hemorrhage, and inhibitors of ferroptosis can reduce neuron degeneration and ameliorate neurologic deficits induced by ischemic stroke and intracerebral hemorrhage (Tuo et al., 2017; Karuppagounder et al., 2018; DeGregorio-Rocasolano et al., 2019). Emerging studies have revealed a tight connection between ferroptosis and VCI, suggesting a potential theoretical approach to treat it. We will describe the possible mechanism of ferroptosis in VCI.

## Iron and VCI

Aging is the main risk factor for dementia, and iron progressively accumulates in the brain during aging. Neuronal iron deposits are closely related to neurodegeneration and cognitive impairment, but the mechanisms underlying these associations remain unclear (Zecca et al., 2004). Recent studies have found that intracellular iron retention and iron-dependent lipid ROS accumulation are the key to trigger ferroptosis, which provides new therapeutic approaches for multiple diseases (Dixon and Stockwell, 2014; Xie et al., 2016). Intracellular iron homeostasis is a complex program involving a series of molecules. DMT1 is the main iron absorption transporter, and FPN1 is the only known protein for iron exportation. The expression of DMT1 and FPN1 was affected by inflammation and aging. Under the stimulation of inflammatory factors (IL-6 and TNF-α), the expression of DMT1 increased and the expression of FPN1 decreased, which resulted in the increase of iron uptake and the decrease of iron excretion in the central nervous cells, resulting in the deposition of iron in the cells (Urrutia et al., 2013). Intracellular excessive iron can induce a large amount of ROS through Fenton reaction or Haber–Weiss reaction, initiating neuronal ferroptosis and resulting in cognitive impairment (Gaasch et al., 2007; Ke and Qian, 2007). Pro-inflammatory cytokines increase due to microglias and astrocyte activation in ischemic stroke, leading to abnormality of iron-related proteins (hepcidin), and brain iron deposition occurs (Petrova et al., 2016). Bilateral common carotid artery occlusion is the most widely used experimental model of VD, and iron deposition leads to neuronal loss caused by oxidative stress, which plays an important role in cognitive impairment of CCH. The most serious neuronal death occurred in the CA1 where the most iron deposits were observed (Li et al., 2012; Du et al., 2017), brain iron dyshomeostasis and iron deposition are closely related to cognitive impairment, and iron-induced ferroptosis has been proved to play an important role in neurodegenerative diseases such as AD, PD, and HD (Ward et al., 2014; Ayton et al., 2017, 2019). The research showed that abnormal iron deposition occurred in a wide range of cortical areas in patients with subcortical ischemic VD, resulting in neuronal damage, which was closely related to the severity of cognitive impairment (Liu et al., 2015). The model of cerebral ischemia–reperfusion injury confirms that proferroptotic iron accumulation is a novel mechanism of injury in stroke, leading to neuronal death. The application of ferroptosis inhibitor (Fer-1, liproxstatin-1) significantly reduced the infarct volume and prevented ongoing neuronal damage, and iron chelators (DFO, a ferroptosis inhibitor) attenuate ischemic–reperfusion damage in animal models (Tuo et al., 2017). It is indicated that iron-induced ferroptosis is a potential mechanism of neuronal loss in VCI.

## Lipid Peroxidations and VCI

Oxidative stress resulted from hypoperfusion has been proved to be one of the main pathogenic mechanisms causing VCI (Jellinger, 2013; Zhang T. et al., 2017), and the study shows that VD patients expressed significantly higher levels of lipid peroxidation markers (MDA) than AD, which suggests that lipid peroxidation has an important impact on the pathophysiology of VD – the MDA level is a possible marker for VD (Gustaw-Rothenberg et al., 2010). Lipid peroxidations and ROS accumulation are the key procedures to induce ferroptosis (Dixon and Stockwell, 2014). LOX can cause lipid peroxidation by catalyzing polyunsaturated fatty acids in phospholipid membrane, and inhibition of LOX can inhibit ferroptosis (Kagan et al., 2017; Shah et al., 2018). After global and focal cerebral ischemia, the widespread increase of 12/15-LOX in brain tissue is an important cause of neuronal cell death and nerve function damage, and inhibition of 12/15-LOX reduced neuronal cell death and the degrees of cerebral edema and improved neurological outcome (Jin et al., 2008; Pallast et al., 2010; Yigitkanli et al., 2017). In addition, nicotinamide adenine phosphate dinucleotide (NADPH) oxidase (NOX) also plays an important role in lipid peroxidation. It is shown that the expression of NOX1 in hippocampal neurons increases during CCH, which leads to lipid peroxidation and oxidative stress. It is an important cause of hippocampal neuronal degeneration and cognitive impairment (Choi et al., 2014). Lipid peroxidation caused by NOX is also one of the links of ferroptosis. Nox1 inhibitors showed a different effect in erastin-induced ferroptosis of Calu-1 cells and HT-1080 cells, which is partially effective in HT-1080 cells. It indicates that NOX contributes different proportions to ferroptosis in different cell types (Dixon et al., 2012; Xie et al., 2016). ACSL4 is responsible for the esterification of CoA to free fatty acids in an ATP dependent manner, and then AA- and AdA-containing PE species are the preferred substrates for oxidation. ACSL4 thereby sensitizes to ferroptosis by specifically esterifying AA and AdA into PE. Recent studies show that thiazolidinedione [e.g., rosiglitazone (ROSI)], a drug for the treatment of diabetes mellitus, can selectively inhibit the activity of ACSL4 and then inhibit ferroptosis (Doll et al., 2016; Angeli et al., 2017). Studies have shown that ACSL4 is widely expressed in the brain tissue, especially in the CA1 region of the hippocampus, and the expression of ACSL4 increases gradually during cerebral ischemia (Cao et al., 2000; Gubern et al., 2013). It has been proved that ROSI can reduce lipid peroxidation and oxidative stress damage in hippocampal neurons during CCH and protect brain function (Sayan-Ozacmak et al., 2012). Multiple studies have shown that long-term administration of pioglitazone can reduce the risk of dementia in patients with non-insulin-dependent diabetics (Heneka et al., 2015; Lu et al., 2018). These links suggest that ferroptosis is a possible mechanism of neuronal loss in CCH, which leads to VCI.

## Glutathione Metabolism and VCI

Thus far researchers have demonstrated that amino acid metabolism is involved in ferroptosis. GPX4 is the sole enzyme for scavenging lipid oxygen free radicals by reducing lipid peroxides to non-toxic lipid alcohols (Lei et al., 2019). GPX4 is a central regulator of ferroptosis; once GPX4 is inactivated, lipid peroxides gradually accumulate, which is identified as the executive signal of ferroptosis (Yang W. S. et al., 2014). And GSH, an essential cofactor, is an important element for GPX4 activity. GPX4 must use GSH as a substrate to eliminate intracellular lipid ROS and maintain redox equilibrium. Thus, GSH depletion disarranges the equilibrium of antioxidant defense and induces ferroptotic cell death (Doll and Conrad, 2017). The system xc-provides the substrate for the synthesis of GSH by transporting cystine into the cell. When the function of system xc- or GPX4 is impaired, lipid peroxide and its degradation products accumulate and induce ferroptosis (Ingold et al., 2018). CCH could result in excessive glutamate released by the depolarization of neurons and occur excitotoxicity, and high levels of glutamate inhibit the function of system xc-. And therefore glutamate excitotoxicity is also a pathomechanism of ferroptosis, and iron chelation prevented the excitotoxic cell death (Krzyzanowska et al., 2014; Liu et al., 2016; Fricker et al., 2018).

The nuclear factor erythroid 2-related factor 2 (NRF2) is a fundamental regulator of cell antioxidant defense system, which modulates the expression of multiple antioxidant response element-dependent genes including NADPH-quinone oxidoreductase 1 (NQO1), heme oxygenase-1 (HMOX1), ferritin heavy chain 1 (FTH1), FPN1, GSH, and GPX4 (Hybertson et al., 2011; Ma, 2013; Kerins and Ooi, 2018). These downstream genes’ expression of NRF2 plays an important role in the ferroptosis signal pathway, and research has showed that the expression level of NRF2 was directly related to the sensitivity of ferroptosis. The increased expression of NRF2 inhibited ferroptosis, and the decreased expression of NRF2 promoted ferroptosis (Sun et al., 2016; Fan et al., 2017; Dodson et al., 2019). Studies have shown that, on the one hand, NRF2 promotes the expression of GSH and GPX4 to enhance the function of antioxidant system, while on the other hand, NRF2 can also reduce intracellular iron accumulation by promoting the expression of ferritin and FPN1 to store and export free iron simultaneously, thereby preventing ferroptosis (Yang X. et al., 2017; Kasai et al., 2018). Many studies have attested to the NRF2 regulatory network playing a fundamental role in different cerebral ischemia rodent models. Although the expression of NRF2 is controversial in different studies, the neuroprotective effect of enhancing Nrf2/ARE activation has been proved in various studies (Zhang R. et al., 2017; Liu et al., 2019). Our previous research and other teams found that the increased expression of NRF2 can ameliorate cognitive impairment in CCH (Yang Y. et al., 2014; Qi et al., 2018; Mao et al., 2019). We speculate that it may also be related to the suppression of ferroptosis, and the GSH metabolic network is the bridge to link ferroptosis and VCI.

## Summary and Outlook

With further research in the field of cell death, so far 12 regulated cell deaths (RCDs) have been defined by the Nomenclature Committee on Cell Death (NCCD) from different perspectives (morphologically, biochemically, and functionally) (Galluzzi et al., 2018; Tang et al., 2019). Ferroptosis is a non-apoptotic form of RCD driven by the iron-dependent accumulation of toxic lipid ROS, which involves many human diseases, especially neurological diseases such as AD, PD, stroke, and intracerebral hemorrhage (Stockwell et al., 2017). For now, researchers have shown that ferroptosis contribute to neuronal loss of acute brain injury, and inhibition of ferroptosis could reduce cell death and ameliorate the neurological function in animal models (Magtanong and Dixon, 2019). But ferroptosis still requires further study in CCH, the main pathological mechanism of VCI. Indeed, the characteristics of ferroptosis are consistent with the pathophysiology of CCH. In summary, an improved understanding of the ferroptosis mechanism and the role of ferroptosis in CCH will create new opportunities for VCI diagnosis and therapeutic intervention.

## Author Contributions

NY drafted the manuscript. JJ-Z revised the manuscript. Both authors read and approved the final manuscript.

## Conflict of Interest Statement

The authors declare that the research was conducted in the absence of any commercial or financial relationships that could be construed as a potential conflict of interest.
